# Disordered Maternal and Fetal Iron Metabolism Occurs in Preterm Births in Human

**DOI:** 10.1155/2022/1664474

**Published:** 2022-08-22

**Authors:** Wei Liu, Yue Wu, Na Zhang, Sijin Liu, Li Zhou

**Affiliations:** ^1^State Key Laboratory of Environmental Chemistry and Ecotoxicology, Research Center for Eco-Environmental Sciences, Chinese Academy of Sciences, Beijing 100085, China; ^2^University of Chinese Academy of Sciences, Beijing 100049, China; ^3^Beijing Obstetrics and Gynecology Hospital, Capital Medical University, Beijing 100026, China; ^4^Beijing Maternal and Child Health Care Hospital, Beijing 100026, China

## Abstract

**Background:**

Increasing evidence reveals that iron deficiency during pregnancy causes adverse pregnancy outcomes. Thus far, the mechanisms underlying iron deficiency-associated preterm birth are mostly limited to animal studies. Whether the suggested mechanisms exist in human requires further investigation. The goal of this study was to characterize the iron metabolism in both the maternal side and fetal side in pregnant women with preterm birth.

**Methods:**

Serum and placenta samples were collected from 42 pregnant women divided into four groups according to the gestational week. Indicators of iron metabolism, including serum iron, serum hepcidin, placental tissue iron, ferroportin (FPN), transferrin receptor 1 (TfR1), and ferritin, were surveyed using enzyme-linked immunosorbent assays (Elisa), Western blots, and real-time quantitative polymerase chain reactions (qRT-PCR).

**Results:**

Significant reduction of maternal serum iron was observed in women with preterm birth relative to those with full-term birth, indicative of worsen iron deficiency in those mothers with preterm birth. Meanwhile, the maternal hepcidin levels were notably diminished in women with preterm birth, whereas the fetal hepcidin levels were comparable between the two groups. Moreover, the placental iron stores were remarkably reduced in the preterm group, associated with reduced concentration of TfR1 and increased FPN concentration relative to the normal controls. In other words, the ratio of placental FPN mass to TfR1 mass (PIDI index) was strikingly increased in the preterm group.

**Conclusions:**

Dysregulated iron homeostasis in both the maternal and fetal sides was implicated in preterm births, and disordered regulations in maintaining the placental iron equilibrium were also presumed to account for the compromised fetal iron supply.

## 1. Introduction

Preterm birth defined as delivery at <37 weeks of gestation is a complex syndrome with uncertain causes [1]. Preterm birth considerably increases the risk of neonatal morbidity, mortality, and long-term sequelae [2–4]. Most preterm births occur spontaneously except for medical or nonmedical reasons where labor is induced or cesarean birth is required [5]. The etiology of preterm births could be attributed to various mechanisms, including infection, progestogens, potential multigenerational effects, preconception and early-pregnancy lifestyle, depression and posttraumatic stress disorder, abnormal allograft reaction, and placental insufficiency [6–12]. In fact, multiple risk factors are assumed to be intertwined to dictate the occurrence of preterm labor.

Iron is a necessary metal for nearly all species and is involved in a broad range of biological processes, including oxygen transport, electron transport, and DNA synthesis [13, 14]. In pregnancies, iron transported through the placenta from the mother to the fetus is critical for embryonic development and fetal growth. The maternal iron demand elevates dramatically to meet the expansion of the maternal blood volume, placental function, and fetal growth [15]. Iron deficiency during pregnancy has been associated with severe adverse pregnancy outcomes, including increased maternal mortality, maternal illness, perinatal death, and preterm birth [16–18]. Moreover, suboptimal iron availability at birth afflicts cognitive behavioral and motor skills and otherwise increases the risk of anemia in infancy [19]. The iron availability for the fetus is subject to the regulation by iron stores in the fetal liver and placenta, placental iron transportation, and maternal iron supply. Since mammals per se harbor no capability to synthesize and secrete iron, sufficient iron absorption from dietary sources is extremely important for both the mother and fetus [20, 21]. To this end, hepcidin, the master regulator of systemic iron metabolism, is downregulated in pregnancies to enhance dietary iron absorption and iron redeployment from storage sites (such as macrophages) by elevating the activity of ferroportin (FPN), the receptor of hepcidin as the sole iron exporter in mammals [22]. Despite recent encouraging progress on iron transport between the maternal side and the fetal side, the molecular basis that account for the dysregulated iron metabolism in abnormal pregnancies has not been thoroughly elucidated [16]. For example, little is known on the iron states in the mother, the placenta, and the fetus, and how changes in the crucial regulators responsible for iron transport contribute to preterm births.

To address the above questions, in the current study, we collected human serum samples and placental specimens at delivery from women with preterm birth. Collectively, we uncovered disordered iron metabolism in both the maternal side and the fetal side during preterm birth, plus the interface inbetween, the placenta. Our findings provide new insights into the mechanisms underlying the human premature births from the angle of iron regulations.

## 2. Materials and Methods

### 2.1. Sample Collection and Grouping

Human serum samples were collected from pregnant women who visited the Beijing Obstetrics and Gynecology Hospital between June 2020 and December 2020. The samples were divided into four groups according to the gestational week: (i) participants in the first trimester (pregnancy weeks < 12), (ii) participants in the second trimester (weeks 13 to 27), (iii) participants in the third trimester (weeks 28 to birth at weeks > 37) with a normal delivery at term, and (iv) participants in the third trimester who experienced the delivery less than 37 weeks of gestational time (preterm). All serum samples were collected from the hospital laboratory as residual specimens and would be discarded after the planned assays. The cord blood samples (5 from Group (iii) and 13 from Group (iv)) and placenta tissues (9 from Group (iii) and 7 from Group (iv)) were collected after delivery. Participants diagnosed with any other maternal complications, such as obesity, gestational diabetes, preeclampsia, and perinatal inflammation, were excluded. The current research was approved by the ethics committee at the Beijing Obstetrics and Gynecology Hospital, Capital Medical University (with the approval number: 2020-KY-033-01). Informed consent was obtained from all participants before sample collection. The research was approved by the Ethics Committee at the Research Center for Eco-Environmental Sciences, Chinese Academy of Sciences.

### 2.2. Clinical Data Collection

The demographic information of all participants was obtained through medical records, including hematological indices, age, BMI, and gestation. All the information was collected at the moment of sampling.

### 2.3. Assays of Serum Biochemical Parameters

Serum iron levels were measured with a serum iron assay kit according to the manufacturer's instructions (Nanjing Jiancheng Bio-Engineering Institute, China). Serum hepcidin levels were assessed using enzyme-linked immunosorbent assay (Elisa) following the manufacturer's protocol (Cloud-Clone Corp., China).

### 2.4. Assay for Tissue Iron

Placenta specimens were ultrasonicated in an ultrasonic instrument with cubic zirconia beads and were then incubated in the acid solution (49.6% HCl, 20% saturated trichloroacetic acid, 30.4% H_2_O, *v*/*v*/*v*) at 65°C for 72 hours. Iron concentration of each sample was measured by Chromagen solution. The absorbance at 535 nm was recorded with a Varioskan™ Flash multimode reader (Thermo Fisher Scientific, USA).

### 2.5. Western Blotting

Placenta tissues were lysed through homogenization in RIPA buffer (Applygen, China), and the obtained lysates were cleared by centrifugation at 12,000 g for 10 minutes at 4°C. Total protein concentrations were quantified by the bicinchoninic acid (BCA) assay kit (Solarbio, China). Thereafter, equal amounts of protein for each sample were loaded and subjected to sodium dodecyl sulfate-polyacrylamide (SDS-PAGE), followed by transfer onto nitrocellulose membranes. The blots were blocked with 5% bovine serum albumin (BSA) in tris-buffered saline with 0.1% Tween 20 (TBST) for one hour at room temperature. The membranes were incubated overnight with primary antibodies (Abs) as follows: rabbit anti-ferroportin (FPN) (a generous gift from Prof. Greg Anderson, QIMR Berghofer Medical Research Institute, Australia), rabbit anti- transferrin receptor 1 (TfR1) (Proteintech, China), rabbit anti-ferritin light chain (FT-L) (Proteintech, China), and rabbit anti-*β*-actin (ABclonal, China). After incubation with the HRP-conjugated secondary Abs, specific bands were visualized through chemiluminescent imaging using an enhanced chemiluminescence (ECL) kit (Thermo Fisher Scientific, USA) on the ChemiDoc XRS+ system (Bio-Rad, USA). The values of FPN, TfR1, and FT-L masses were normalized to the loading control, *β*-actin. The PIDI index was calculated by dividing the FPN values by those of TfR1 values, as reported [16].

### 2.6. Real-Time Quantitative Polymerase Chain Reactions

Total RNA was isolated using TRIzol (Thermo Fisher Scientific, USA), and cDNA was synthesized using a reverse transcription kit (Forever Star Biotech, China). Real-time quantitative polymerase chain reaction (qRT-PCR) assays were set up by GoTaq qPCR Master Mix according to the manufacturer's instructions (Promega, USA). The samples were analyzed using a CFXconnect qPCR instrument (Bio-Rad, USA). Primers were designed for target gene (human *REG-1*) (forward TCCTGCGTAAGAAGCCACTCAC, reverse GGTGGAAGAATCGGCACTTGATC) and the reference gene (human *β-actin*) (forward primer CACCATTGGCAATGAGCGGTTC, reverse primer AGGTCTTTGCGGATGTCCACGT). Calibrations were done using the ΔCt method, where ΔCt = (Ct (target gene) − Ct (reference gene)).

### 2.7. Statistical Analyses

Statistical analyses were performed using GraphPad Prism 8.0.2 (GraphPad Software Inc.). The data are presented as the mean ± standard deviation (SD). Comparisons were performed with the 2-tailed Student's *t*-test for normally distributed values, and with the Mann–Whitney *U* test for nonnormally distributed values. One-way ANOVA with the Tukey means comparison test or a 2-way ANOVA was used to determine the group differences. The numbers of individuals in each group are indicated in according panels. *P* values of less than 0.05 were determined statistically significant.

## 3. Results

### 3.1. Demographics and Clinical Characteristics of Pregnant Women

**Figure 1:**
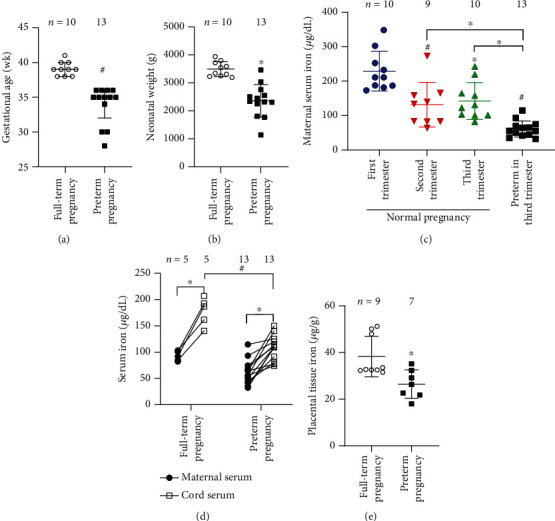
Changes of iron-related indices in premature pregnancies. Gestational age (a), neonatal weight (b), maternal serum iron (c), serum iron (d), and placental tissue iron (e). ^∗^*P* < 0.05 and ^#^*P* < 0.001, compared to the full-term pregnant control or as indicated.

To exclude the possible confounders as much as possible such as different gestational age, altered systemic inflammation, obesity, and other complications, samples of participants without maternal complications were collected, and then, we focused our analysis on iron metabolism. Important information was collected from the four groups of participants (Table 1). Demographic characteristics showed comparable average age and body mass index (BMI) among the groups, and the complete blood count (CBC) data, including red blood cells (RBCs), hemoglobin content (HGB) did not recognize significant differences. Inflammation has been demonstrated to elevate hepcidin production via the interleukin 6 (IL-6)/signal transducer and activator of transcription 3 (Stat3) signaling pathway to modulate the systemic iron metabolism, where inflammation is induced by bacterial or viral infection, obesity, and diabetes during pregnancy [23]. In the present study, the WBC counts (×10^9^/L), neutrophils (%), and lymphocyte (%) were comparable among the groups, suggesting that inflammation may not be account for the regulation of hepcidin in these women with or without preterm pregnancies. Moreover, no differences were defined for levels of vitamins A and E, and the percentages of iron supplementation were slightly greater in the preterm group than that in the other groups. Regardless, the average gestational period was shorter at 33.92 weeks in the preterm group (refer to information or specimens collected from mothers with preterm birth in the third trimester or at the point of delivery) than that in the full-term group (refer to information or specimens collected from normal mothers in the third trimester or at the point of delivery) at 39.10 weeks (Figure 1(a)), *P* < 0.001). The average preterm neonatal weight (2,360 g) was much lighter than the average full-term neonatal weight (3,493 g) (Figure 1(b)), *P* < 0.001).

### 3.2. Iron Metabolism in Premature Pregnancies

Next, we scrutinized iron homeostasis in women with full-term and preterm deliveries. The cord blood samples and placental tissues were collected after delivery. In analogy to previous findings, the maternal serum iron concentrations largely declined in the normal pregnancies from the first trimester to the third trimester (Figure 1(c)), *P* < 0.05), associated with the increased fetal iron demand [24, 25]. And the enlarged volume of the total blood during the gestation may also dilute the iron concentrations [26, 27]. In this study, we found that serum iron levels were lower in participants sampled in the third trimester than those sampled in the first trimester (Figure 1(c)), *P* < 0.05). Nevertheless, similar to previous results [28], maternal serum iron content was further reduced in participants with preterm birth, as reflected by the 57.00% drop relative to women with full-term births in the third trimester (Figure 1(c)), *P* < 0.001), suggestive of the worsening of iron deficiency in women with preterm outcome in spite of high rate of iron supplementation in this group (Table 1). To examine the changes of iron levels in more details, we compared iron concentrations in the cord blood and corresponding maternal blood, namely, paired comparison between cord blood and maternal blood in both full-term and preterm pregnancies. In participants with full-term births, the serum iron levels were greatly increased by more than 90% in the cord blood in comparison to that in the maternal blood (Figure 1(d)), *P* < 0.05) implying considerable demand for iron during fetal development [29, 30]. By contrast, the increase in iron concentration in the cord blood relative to the maternal blood was only 77.20% in participants with preterm births, smaller than that in the full-term births (line connecting the mother and corresponding child) (Figure 1(d)), *P* < 0.05). Likewise, the iron concentrations in the cord blood were much lower than that in full-term births, revealing remarkable iron deficiency in the developing fetuses prior to preterm births (Figure 1(d)), *P* < 0.001). Previous animal studies have portrayed the placenta as a selfish organ since the placenta stores an adequate amount of iron to ensure its functions in maternal iron deficiency [13]. However, a recent study conducted by Irwinda et al. indicated that the placental iron stores in women with preterm births were much lower than that in women full-term births [28]. Besides, placental iron content in humans was reported ~50% lower than previously estimated in animal studies, and the placental iron concentrations were significantly associated with the maternal iron status, indicating that distinct mechanisms may be involved in different species [31]. In our work, in agreement with reduced iron concentrations in maternal and cord blood, the placental iron stores were reduced by >30% in the preterm group, compared to that in the full-term group (Figure 1(e)), *P* < 0.05). The lower iron concentrations in the cord blood from fetuses prior to the preterm birth were presumably caused by the worsened maternal iron deficiency.

### 3.3. Changes of Iron Regulators in Preterm Labor

**Figure 2:**
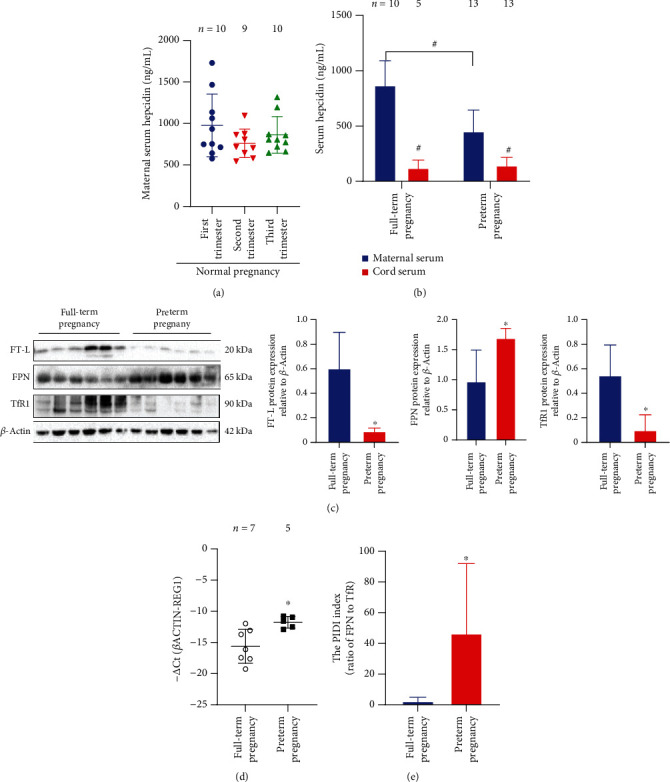
Changes of maternal and cord serum hepcidin and iron regulators in preterm pregnancies. Maternal serum hepcidin at different stages (a), maternal and cord serum hepcidin (b), the PIDI index (c), Western blot analysis of FT-L, FPN, TfR1, and *β*-actin (d); the quantified data for Western blot results are presented in the right panel, relative *REG-1* expression (e). ^∗^*P* < 0.05 and^#^*P* < 0.001, compared to the full-term pregnant control or as indicated.

To shed light on the molecular bases underlying iron deficiency on both maternal and fetal sides in preterm births, crucial regulators of iron metabolism were therefore assessed. As shown in Figure 2(a), the maternal hepcidin concentrations in women with full term births dropped by more than 22% after the second trimester, compared to that in the first trimester. This result is analogous to the previous findings that maternal hepcidin levels are diminished to enhance dietary iron absorption and mobilization for the increasing iron demand from the developing fetus [32, 33]. Moreover, the maternal hepcidin levels were further decreased by 47.73% in participants with preterm births relative to that with full-term births (Figure 2(b)), *P* < 0.001), presumably due to more severe iron deficiency in women with preterm births. Emerging evidence uncovered a critical role of fetal hepcidin in the regulation of fetal iron utilization in fetal liver erythropoiesis [19]. Under this context, the hepcidin level in the cord blood was also determined, as shown in Figure 2(b). Similar to the recent studies, the fetal hepcidin levels were much lower than the maternal hepcidin levels (Figure 2(b)), *P* < 0.001), which may support the autonomous modulatory mechanism for iron mobilization in the fetus. Intriguingly, the fetal hepcidin levels in cord blood from those with preterm births were comparable to that with full-term births (Figure 2(b)), *P* = 0.95), suggesting already rather low level of hepcidin in the fetal stage. This observation thus pinpointed the crucial role of maternal hepcidin in regulating iron homeostasis in the mother and placenta [34, 35].

### 3.4. Dysfunction of Placental Iron Transportation in Premature Births

In the following, the placental iron stores and regulators of iron transport were explored. Greatly decreased placental iron stores were found in participants with preterm births relative to those with full-term births as evidenced by decreased placental tissue iron (Figure 1(e)), *P* < 0.05) and diminished mass of ferritin (Figure 2(c)), *P* < 0.05). Despite no direct experimental evidence, the maternal hepcidin is assumed to control the placental iron transport through syncytiotrophoblasts [13, 36, 37]. The mass of FPN in placentas was greatly elevated in the preterm group, compared to the full-term group (Figure 2(c)), *P* < 0.05) presumably attributable to the diminished maternal hepcidin levels (Figure 2(b)), *P* < 0.001), implying a direct regulation of maternal hepcidin on the FPN concentrations in syncytiotrophoblasts. In the meantime, the expression of transferrin receptor 1 (TfR1) was greatly reduced in placentas from the preterm group relative to that in the full-term group (Figure 2(c)), suggesting that reduced TfR1 indeed impaired the uptake of iron into syncytiotrophoblasts. The endonuclease regnase-1 (REG-1) has been proved to play an important role in cellular iron homeostasis via destabilizing the mRNA of iron-regulating genes, such as TfR1 [38, 39]. Under this context, we further assessed the REG1 expression at the transcription level. As shown in Figure 2(d), the REG1 mRNA expression was greatly induced in placentas from participants with preterm births, which might partially account for the decreased TfR1 expression. Next, we calculated the ratio of placental FPN mass to TfR1 mass, namely, PIDI index [16]. Here, we observed greatly increased PIDI in the preterm group, with more than 19-fold increase relative to the full-term group (Figure 2(e)). Thereby, the reduced placental iron stores could be ascribed to compromised TfR1 levels and otherwise increased FPN levels.

## 4. Discussion

Iron is crucial for both mother and fetus during gestation. Due to the essential role of iron in development, iron deficiency can retard the early development, compromise cognitive ability of children, and increase the likelihood of an abnormal pregnancy. Conversely, iron overload otherwise can damage cells and tissues being ascribed to the chemical reactivity of iron and its ability to generate reactive hydroxyl radicals through the Fenton reaction to eventually oxidize lipids, proteins, and DNA. Therefore, mammals have evolved sophisticated mechanisms to maintain appropriate iron concentrations, including the hepcidin-FPN axis, iron regulatory proteins (IRPs)/iron responsive element (IRE) system, and hypoxia inducible factor-2*α*- (HIF2*α*-) mediated transcriptional regulation [40].

In our study, the dramatically reduced cord serum iron levels in the preterm group should be attributable to the lower maternal serum iron levels, indicating that women with premature births were experiencing iron deficiency during pregnancy (Figures 1(c) and 1(d)). As an important self-protection mechanism to maintain iron homeostasis, hepcidin suppression during iron deficiency allows for the redeployment of the body iron stores [41]. We found that maternal hepcidin levels were decreased in participants with preterm births (Figure 2(b)), implying that the preterm mothers were endeavoring to correct the undergoing iron deficiency. Meanwhile, the placenta is an important organ for iron storage and plays a crucial role in supplying iron to the fetus throughout pregnancy to support fetal growth and development. In maternal iron deficiency, the placenta would give the priority to maintain sufficient iron stores to support its functions and to deliver iron to the fetus despite the low iron levels in maternal blood [13]. However, in analogy to a recently reported study [28], the placental iron stores were apparently compromised under preterm condition (Figure 1(e)). These results indicate that distinct mechanism may account for the human preterm-related iron deficiency, different from animal model with pure iron deficiency condition [31], which warrants detailed studies to address the discrepancies further.

During cellular iron deficiency, IRPs bind to IREs within the untranslated regions (UTRs) of iron-related genes. The binding of IRPs to the 5′ IREs prevents the translation of mRNAs responsible for iron storage and export (such as ferritin and FPN) [15]. As observed in the current work, the FPN level in placentas from the preterm group was increased (Figure 2(c)), presumably due to the lower maternal hepcidin levels. These data also hinted at a protective mechanism for the promoting of iron transport into the fetus despite the iron deficit in the mothers, which warrants further detailed investigations. In rats with maternal iron deficiency, placental TfR1 and divalent metal transporter (DMT1) expression were increased due to the binding of IRPs to 3′ IREs enhancing the stabilization of mRNAs for increased iron requirement [16, 42]. Nevertheless, unlike the pure iron-deficient animal model, TfR1 level on the apical surface of syncytiotrophoblasts was reduced in participants with preterm births (Figure 2(c)). Since previous studies have demonstrated that REG-1 could destabilize the mRNA of TfR1 [39], we would speculate that the decreased placental TfR1 in preterm group might be attributed to the induced REG-1 expression (Figure 2(d)) or due to other unknown mechanisms in preterm births. As reported previously, the PIDI was generated to characterize the iron efflux to the fetal side relative to iron influx into the placenta from the maternal side. Increased PIDI would reflect reinforced iron export from the maternal blood circulation into the fetus [16]. Our current data showed that PIDI was significantly elevated under preterm circumstance (Figure 2(e)), in stark contrast to the study by Sangkhae et al. [16]. These results collectively unraveled that the iron transportation within placentas in the preterm group was disordered. This difference in results could be attributed to the distinct mechanisms in different models, as iron deficiency is an important but not the only contributing factor in preterm births.

Besides, the HIF system is another crucial regulator of transcription for genes important in iron metabolism. During iron deficiency, increased hypoxia or erythropoietic drivers induce HIF2*α* expression in the duodenal epithelium [43]. Since there are hypoxia-responsive elements (HREs) in the promoters of FPN1, DMT1, and duodenal cytochrome b (DCYTB), HIF-2*α* has been found to transcriptionally regulate the expression of these iron transporters in iron deficiency and anemia [44]. However, it is currently unclear whether a similar mechanism exists in placental tissues in preterm births. Additionally, we have to admit that the differences in developmental age of the placenta, the placental tissue integrity between normal and preterm birth, and other unknown preterm pathologies (which cannot entirely be avoided) may also have a significant influence on the outcomes.

## 5. Conclusions

Although current findings suggest that iron deficiency during pregnancy is associated with detrimental outcomes, the according mechanisms underlying the occurrence of preterm births have not yet been fully elucidated, especially in humans. In this study, we found dysregulated iron metabolism in both maternal side and fetal side in pregnant women with premature births, as identified by the declined maternal and fetal serum iron levels. Investigations into the mechanisms revealed diminished maternal hepcidin levels in women with preterm births, corroborating iron deficit in the mothers and hinting a strong influence on the placental interface. Consistently, reduced iron stores and together with increased FPN expression were found in the placental tissues from women with preterm delivery. In the meantime, placental TfR1 expression was reduced in preterm births, which might be partially accountable for the compromised placental iron stores. The reduced TfR1 expression could be attributed to the downregulation by the increased REG-1. Notably, the PIDI was strikingly increased in the preterm group, which should differ from the pure iron deficiency condition. Together, the disordered regulations along the cascade from the mother, to the placenta and the fetus, would impair iron supply and eventually undermine the fetal growth and development, resulting in increased occurrence of preterm births. Nevertheless, despite these findings, the detailed regulatory mechanisms warrant further investigations in the future. This study would open a new path to understand the etiology of preterm births from the perspective of iron metabolism.

## Figures and Tables

**Table 1:** Demographic parameters of the enlisted subjects.

Parameters	Normal pregnancy			Preterm
	First trimester (*n* = 10)	Second trimester (*n* = 9)	Third trimester (*n* = 10)	Third trimester (*n* = 13)
Age (y)	30.00 ± 2.94	29.22 ± 3.38	31.40 ± 4.20	30.38 ± 2.18
BMI (kg/m^2^)	21.86 ± 1.74	20.60 ± 1.78	234.38 ± 4.03	21.33 ± 3.66
RBC (×10^12^/L)	4.22 ± 0.25	3.82 ± 0.29	4.03 ± 0.26	4.07 ± 0.31
HGB (g/L)	129.60 ± 4.84	121.20 ± 7.58	129.40 ± 9.42	126.00 ± 8.30
WBC (×10^9^/L)	8.29 ± 1.56	9.57 ± 2.03	8.96 ± 2.04	10.32 ± 1.72
Neutrophils (%)	73.40 ± 5.52	70.57 ± 4.35	73.34 ± 4.53	72.48 ± 5.45
Lymphocyte (%)	20.95 ± 4.46	22.40 ± 3.75	19.18 ± 4.99	19.59 ± 5.04
Vitamin A (mg/L)	—	0.44 ± 0.09	0.42 ± 0.09	0.47 ± 0.11
Vitamin E (mg/L)	—	15.06 ± 3.02	15.68 ± 4.04	14.42 ± 2.26
Iron supplement, *n* (%)	1 (10%)	1 (11.11%)	1 (10%)	3 (23.08%)

BMI: body mass index; RBC: red blood cell count; HGB: hemoglobin; WBC: white blood cell count. Data are presented as the mean ± SD. No statistically differences were found among these groups.

## Data Availability

The study data presented may be made available from the corresponding author upon reasonable request.
